# Drift-Robust Lightweight Deep Learning on Open Gas Sensor Benchmarks: A Reproducible Architecture Study with CBRN Applicability Mapping

**DOI:** 10.3390/molecules31111884

**Published:** 2026-06-01

**Authors:** Soohwan Kim, Myeongsik Shin, Ku Kang, Doo-Hee Lee, David G. Churchill, Yoon Jeong Jang

**Affiliations:** 1 CBRN Defense Research Institute, Seoul 06796, Republic of Korea; tnghks1930@gmail.com (S.K.); fhwm0448@snu.ac.kr (M.S.); bisu9082@gmail.com (K.K.); dooheeleechem@gmail.com (D.-H.L.); 2 Department of Chemistry, Korea Advanced Institute of Science and Technology (KAIST), Daejeon 34141, Republic of Korea; 3 Therapeutic Bioengineering Section, KAIST Institute for Health Science and Technology (KIHST), Daejeon 34141, Republic of Korea

**Keywords:** CBRN detection, gas sensor array, edge AI, TinyML, knowledge distillation, sensor drift compensation

## Abstract

Resource-constrained edge processors deployed on unmanned aerial vehicles and wearable platforms require compact, drift-robust gas classification models for a range of environmental and security monitoring applications, including CBRN-motivated scenarios. Existing approaches rely on server-grade architectures incompatible with edge-board-scale deployment, or on classifiers that chemically degrade severely under long-term sensor drift. Each UCI gas class was mapped to a CBRN behavioral category based on physicochemical analogy (molecular functional group, vapor pressure, and metal-oxide semiconductor (MOS) cross-sensitivity pattern), following established precedent. Analyzed were Ammonia (NH_3_), Acetaldehyde (CH_3_CHO), Acetone ((CH_3_)_2_CO), Ethylene (C_2_H_4_), Ethanol (C_2_H_5_OH), Toluene (C_6_H_5_CH_3_). We propose herein an end-to-end pipeline integrating a novel 1-D convolutional neural network with depth-wise separable convolutions (LiteSensor-Net), INT8 post-training quantization, structured magnitude pruning, and a knowledge-distillation domain-adaptation module (KD–DM) for sensor drift compensation. Using the UCI Gas Sensor Array Drift Dataset (13,910 measurements; 16 metal-oxide sensors; six analyte gases; a 36-month work span). LiteSensor-Net achieved accuracy = 92.63 ± 2.02%, macro-F1 = 0.898, model size = 5.99 kB INT8 pruned, inference latency = 6.3 ms, RAM footprint = 31.7 kB, and energy per inference = 0.04 mJ (all metrics on Raspberry Pi 4B, ARM Cortex-A72). Under chronological forward-chaining evaluation, KD–DM–20 achieved 47.91 ± 18.79% mean accuracy over Batches 2–10, representing a +9.25 pp improvement over uncompensated NC (38.66%). A six-metric benchmark framework—accuracy, macro-F1, model size, inference latency, RAM footprint, and energy per inference—is introduced to standardize edge-AI gas classifier evaluation. The proposed pipeline provides an open-source, deployable foundation for edge-class gas classification systems, with CBRN detection as a motivating application. Full operational validation on certified chemical simulants remains as future work.

## 1. Introduction

The widespread adoption of unmanned aerial vehicles (UAVs), autonomous ground robots, and soldier-worn wearables in military and disaster-response operations has created an urgent operational requirement for edge-deployed sensor nodes capable of autonomous, high-confidence chemical threat classification [1,2,3]. Chemical, biological, radiological, and nuclear (CBRN) threats represent a persistent and evolving risk category; the demand for platforms that operate without continuous server connectivity—and that can sustain reliable detection over extended field deployments—has therefore become a core constraint in sensor system design [4,5].

Metal–oxide semiconductor (MOS)-based electronic nose (E-nose) systems offer a favorable combination of low cost, compact form factor, fast response time, and broad chemical sensitivity, making them prime candidates for CBRN edge deployment [6,7,8]. However, two structural obstacles have limited their operational maturity. First, the most accurate pattern-recognition architectures—deep ResNets, bidirectional long short-term memory (LSTM) networks, and large ensemble methods—exceed the memory and compute budgets of the single-board-computer-class hardware available on field platforms [9,10,11]. Second, MOS sensors exhibit well-documented long-term drift: resistance baselines shift over months due to progressive degradation of sensor active-layer materials, temperature cycling, and environmental contamination, degrading classification accuracy by 10–30% if left uncompensated [12,13,14].

The prior literature addresses these two challenges in isolation. Model-compression studies benchmark classification accuracy without evaluating long-term drift robustness [15,16], whereas drift-compensation methods—including classifier ensembles [12], knowledge-distillation adaptation [17], and multi-classifier trees [18]—recover accuracy without constraining model size or inference latency [17,18,19], with none reporting deployable footprints compatible with sub-100 kB edge hardware. Conversely, model compression studies [15,20] achieve small footprints but do not evaluate drift robustness. Additionally, evaluations on chemical warfare agent (CWA)-relevant scenarios remain rare in the open-source literature [21,22]. No prior work has simultaneously satisfied sub-100 kB deployability, integrated drift compensation, statistically rigorous multi-metric evaluation, and chronologically honest benchmarking in a single open-source pipeline. The present study addresses these gaps through four integrated contributions.

1. LiteSensor-Net: A custom 1D–CNN employing depth-wise separable convolutions (DSConv), batch normalization (BN), and global average pooling, designed from first principles for sub-6 kB INT8 deployment. Unlike prior 1D-CNN gas-sensing classifiers [9,10,11], LiteSensor-Net is jointly optimized with a drift-compensation module and evaluated under a six-metric edge-deployment benchmark.
2. Multi-stage compression pipeline: Sequential INT8 post-training quantization (PTQ) and structured magnitude pruning achieving 78.64% total model size reduction (28.04 kB → 5.99 kB INT8; 73.7% from INT8 precision scaling, ~5% from structural pruning) with < 0.5% accuracy loss (see Section 2.5).
3. Knowledge-distillation (KD)-based drift-compensation module (KD–DM): A teacher–student domain-adaptation layer that suppresses feature-space drift via KL-divergence regularization, enabling offline server-side adaptation with over-the-air weight updates—without on-device retraining (see Section 2.6).
4. Standardized benchmark framework: A six-metric evaluation protocol enabling transparent, reproducible cross-architecture comparisons (see Section 2.8).

Two limitations are noted at the outset: (i) the UCI-to-CBRN re-labeling is a physicochemical analogy for architectural feasibility demonstration and does not constitute toxicological equivalence to live chemical warfare agents (Section 4.5); (ii) all latency, RAM, and energy benchmarks are measured on a Raspberry Pi 4B (ARM Cortex-A72 SBC); and microcontroller unit (MCU)-class on-device profiling remains a future validation target (Section 5). The remainder of this paper is organized as follows: Section 2 describes the datasets, the simulant re-labeling protocol, LiteSensor-Net architecture, multi-stage compression pipeline, KD-DM, and six-metric benchmark framework. Section 3 presents baseline classification, CBRN simulant classification, and sensor drift compensation results. Section 4 discusses architecture efficiency, drift-compensation mechanisms, benchmark significance, operational reliability, and CBRN applicability. Section 5 is provided in which conclusions are drawn and future work is outlined.

## 2. Materials and Methods

### 2.1. Datasets

#### UCI Gas Sensor Array Drift Dataset (Primary)

The UCI Gas Sensor Array Drift Dataset [12,23] was collected at the ChemoSignals Laboratory, University of California, San Diego, CA, USA, over a period of 36 months (January 2008 to February 2011). The dataset comprises 13,910 steady-state measurements from an array of 16 MOS sensors exposed to six small molecule pure gases—Ammonia (NH_3_), Acetaldehyde (CH_3_CHO), Acetone ((CH_3_)_2_CO), Ethylene (C_2_H_4_), Ethanol (C_2_H_5_OH), and Toluene (C_6_H_5_CH_3_)—at concentrations of 5–1000 ppm. Measurements are organized into ten temporal batches intended to emulate real-world sensor aging (this emulation would involve the chemical degradation of the device; sometimes the phrase “wear and tear” is used). Each observation comprises 128 features (8 steady-state and transient features per sensor: *ΔR*, |*ΔR*|, and 6 exponential moving averages at *λ* ∈ {0.001, 0.01, 0.1} for both increase and decrease phases) [12]. The dataset is licensed under the Creative Commons Attribution 4.0 (CC BY 4.0). All 13,910 measurements were obtained directly from the UCI Machine Learning Repository and used without modification with regard to the original representation features.

### 2.2. CBRN Simulant Re-Labeling Protocol

In this work, no restricted chemical warfare agents (CWAs) were handled. To provide a CBRN-motivated framing for architectural evaluation, each UCI gas class was mapped to a CBRN behavioral category based on physicochemical analogy (molecular functional group, vapor pressure, and MOS cross-sensitivity pattern), following established precedent [1,22]. This mapping is intended solely to demonstrate pipeline feasibility under CBRN-relevant labeling and does not imply toxicological equivalence or validated detection of actual chemical warfare agents. Table 1 details the mapping.

### 2.3. Preprocessing Pipeline

**Figure 1:**
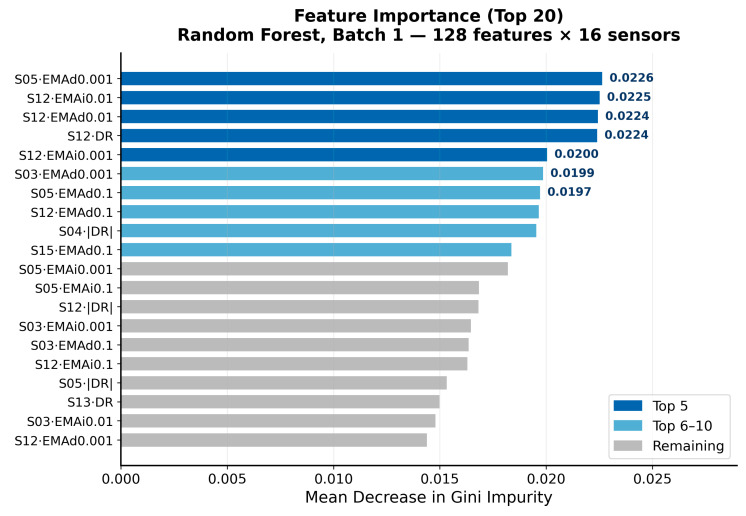
Feature importance ranking (top 20) computed via mean decrease in Gini impurity (Random Forest, *n*trees = 300, Batch 1 training set). Features are labeled as Sensor ID and Feature Type. The |*Δ*R| Feature Type and sensors with high NH_3_ and Toluene sensitivity (S1, S8, S12) dominate the top positions. Blue: top 5; light blue: top 6–10; gray: 11–20.

Four preprocessing stages were applied sequentially:

1. Batch baseline correction: Per-sensor median subtraction within each temporal batch removes slow drift components independent of analyte identity [12].
2. L2 normalization: Each 128-dimensional sample vector is normalized to unit Euclidean length to reduce inter-sensor gain disparities.
3. Feature selection via Gini importance: Random Forest feature importance (scikit-learn ≥ 1.4; 300 trees, Batch 1) ranked all 128 features. The top–*k* = 64 subset was selected after validating that it yields no statistically significant accuracy loss versus *k* = 128 (5-fold cross-validation, *p* = 1.00, Wilcoxon signed-rank test; Figure 1).
4. Standardization: Within-split Z-score normalization (zero-mean, unit-variance) using training-set statistics only.

**Figure 2:**
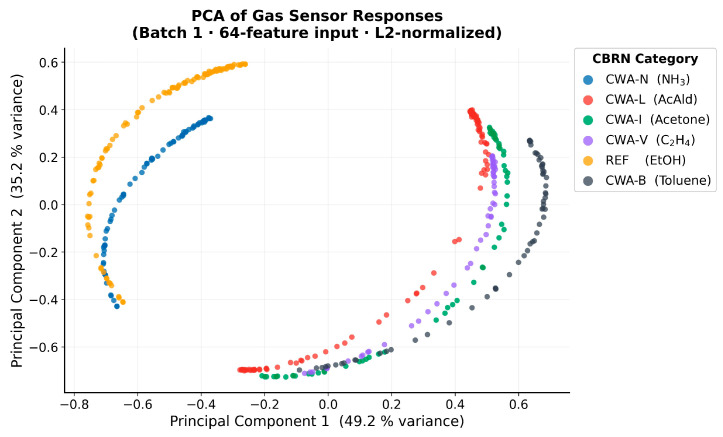
Principal component analysis (PCA) projection of Batch 1 data (64 selected features, L2-normalized) into two principal components (PC1: 49.2%, PC2: 35.2% variance). Each point represents one measurement; colors correspond to CBRN behavioral categories. Well-separated clusters confirm the ability to discern effectively between the different agent classes in the preprocessed feature space.

A principal component analysis (PCA) of Batch 1 (64 selected features) explained 49.2% and 35.2% of variance on PC1 and PC2, respectively (Figure 2), confirming strong between-class discrimination of the preprocessed feature space.

### 2.4. LiteSensor-Net Architecture

**Figure 3:**
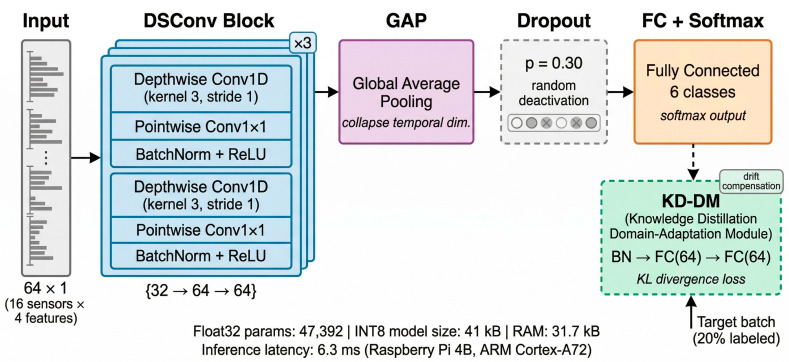
The LiteSensor-Net architecture. Three design constraints were imposed: (i) parameter count < 50,000; (ii) top-1 accuracy (the fraction of test samples whose highest-probability predicted class matches the ground-truth label) ≥ 90% on the UCI primary benchmark; (iii) inference latency < 10 ms on Raspberry Pi 4B.

The network receives a 64-dimensional feature vector reshaped as a 1D sequence (64 × 1). Three depth-wise separable convolution (DSConv) blocks alternate depth-wise convolutions (kernel size 3, stride 1) with pointwise 1 × 1 convolutions, batch normalization (BN), and ReLU activations; channel widths are {32, 64, 64}. The depth-wise operation is applied along the time axis (per-channel temporal filtering), preserving sensor-channel separability at the depth-wise step. Cross-sensor dependencies are restored at the immediately following pointwise 1 × 1 convolution, which acts as a learned linear mixing across all sensor channels. Global average pooling (GAP) collapses the temporal dimension; a dropout layer (*p* = 0.30) and a fully connected (FC) Softmax output follow. The DSConv factorization reduces FLOPs by ≈ 8× and parameters by ≈ 11× relative to a standard ResNet-1D baseline with equivalent receptive field [20,25]. The overall architecture is illustrated in Figure 3.

LiteSensor-Net was trained using the AdamW optimizer with an initial learning rate η_0_ = 3 × 10^−3^, weight decay λ = 5 × 10^−4^, and batch size 16 over 100 epochs. A linear warm-up was applied for the first 10 epochs, followed by cosine annealing to η_min_ = 0. Label smoothing (ε = 0.05) was applied to the cross-entropy loss. An 80:20 training–validation split of the available training data was used for model selection; the checkpoint achieving the highest validation accuracy was retained. Full hyperparameter settings for all pipeline stages are provided in Table S2 (Supplementary Materials).

### 2.5. Multi-Stage Compression

#### 2.5.1. INT8 Post-Training Quantization

Float32 weights and activations were quantized to INT8 using the TensorFlow Lite PTQ pipeline (version ≥ 2.14) [16] with a 512-sample calibration set from Batch 1. Quantization-aware training (QAT) was evaluated but yielded < 0.2% accuracy improvement over PTQ at the cost of 30 min additional training; PTQ was retained for practical simplicity. Under drift conditions, the KD-DM fine-tuning step operates directly on the INT8-quantized student, recalibrating quantized decision boundaries to the shifted target distribution and thereby reducing, but not eliminating, quantization-induced brittleness under covariate shift.

#### 2.5.2. Structured Magnitude Pruning

After quantization, global structured pruning removed channels below the 20th percentile of ℓ1 magnitude scores [26]. The network was fine-tuned for five epochs under a cosine-annealed learning rate (*η*_0_ = 10^−4^). A 20% sparsity target was selected via sweep over {10, 20, 30, 40} % to remain within 1% accuracy loss.

### 2.6. Knowledge-Distillation Drift-Compensation Module

Sensor drift is modeled as a covariate-shift problem [13]: the marginal distribution p(***x***) shifts across temporal batches while the class-conditional distribution p(*y*|***x***_corrected_) remains stable. A KD–DM is appended to the penultimate feature layer of LiteSensor–Net. The teacher is either (i) a single LiteSensor-Net trained on the source batch (KD-DM-single), or (ii) a 3-member ensemble of LiteSensor-Net teachers trained with different random seeds, with logits (or softened output probabilities) averaged before the distillation loss (KD-DM-ensemble). The student remains a single LiteSensor-Net at deployment; ensemble distillation only affects the soft-target supervision signal during fine-tuning. The composite training loss is

L_total_ = *α* L_CE_ (*y*, *ŷ*) + (1 − *α*) *T*^2^ L_KL_ *σ^Zt/T^ σ^Zs/T^* (1)

where L_CE_ is cross-entropy loss on labeled target-batch samples; L_KL_ is the Kullback–Leibler divergence; Zt and Zs are the logits of the teacher and student models, respectively; σ(·) is the Softmax function; T = 4 is the distillation temperature; α = 0.5 balances the two loss terms; y is the ground-truth label; and ŷ is the student’s predicted output. The T^2^ factor compensates for the reduced magnitude of the softened probability distributions [27,28]. The KL-divergence term serves as a regularizing anchor: the teacher’s softened output distribution encodes inter-class similarity structure that is invisible in hard one-hot labels—for example, the relative likelihood of confusing two chemically proximate gases under drift conditions—enabling the student to acquire a richer, drift-robust representation while avoiding catastrophic forgetting of source-domain knowledge. Both α and T were selected by grid search on a held-out validation split of Batch 2 (α ∈ {0.3, 0.5, 0.7}, T ∈ {2, 4, 6}). Domain adaptation uses 20% of the target-batch training partition as labeled fine-tuning data, selected by stratified random sampling per class within each of the five pre-specified splits (seeds: 17, 42, 113, 271, 314); the held-out test partition (30%) is shared across all methods and is never exposed during fine-tuning. This setting simulates a minimal field-calibration scenario in which a small number of labeled samples is available during periodic sensor maintenance. To ensure a resource-equivalent comparison, a modified drift-robust classification and adaptation (DRCA) baseline (DRCA-fair) that receives the identical 20% labeled fraction is also evaluated, alongside a fully unsupervised KD-DM variant (KD-DM-unsup) that derives pseudo-labels from teacher high-confidence predictions (confidence ≥ 0.70). KD-DM operates in an offline server-side topology: target-batch samples are transmitted to a central server during a scheduled maintenance window, teacher–student distillation is executed on float32 weights, and the adapted INT8-pruned student is delivered to the device fleet via secure over-the-air (OTA) update. No training computation is performed on the edge node.

### 2.7. Domain–Adaptation Task Definitions

Two tasks regarding model realistic deployment scenarios are evaluated below [17]. Five conditions are compared: NC (no target-batch data), DRCA (no labeled target data), DRCA-fair (20% labeled target-batch fraction, matched to KD-DM), KD-DM-20 (20% labeled), and KD-DM-unsup (0% labeled, pseudo-label based). This spectrum of conditions allows both scenario-based and resource-equivalent interpretation of results:

- Task A (laboratory re-calibration): Batch 1 trains the model; Batches 2–10 are successive target domains.
- Task B (online continual adaptation): Batches 1, …, *n* − 1 cumulatively train the model; Batch *n* is the target domain.

Task A is evaluated with five pre-specified chronological forward-chaining partitions (seeds: 17, 42, 113, 271, 314). In each partition, the earliest 70% of target-batch samples constitute the adaptation pool, from which 20% of the total target batch (stratified per class) is drawn as labeled fine-tuning data for KD-DM and DRCA-fair. The remaining 30% serves as the held-out test set and is never accessed during adaptation. All random operations are seeded with five pre-specified fixed seeds (17, 42, 113, 271, 314; fully documented in the accompanying code repository), selected prior to any model training and not adjusted based on observed results. Performance statistics (mean ± SD) and pairwise improvements are assessed by two-sided Wilcoxon signed-rank tests at α = 0.05 across the five partitions. Task B is evaluated with 30 independent chronological partitions to provide sufficient statistical power for the cumulative training scenario. The same 70:30 adaptation/test split and 20% labeled fraction apply. Performance statistics are assessed by two-sided Wilcoxon signed-rank tests at α = 0.05 across the 30 partitions.

### 2.8. Evaluation Benchmark Framework

Table 2 is provided below and provides the definition of the six-metric framework evaluated on Raspberry Pi 4B (Raspberry Pi Ltd., Cambridge, UK; ARM Cortex A72, 1.8 GHz, 8 GB LPDDR4).

## 3. Results

### 3.1. Processing and Feature Selection

Figure 2 shows the PCA projection of Batch 1 (64 selected features). The six gas classes form well-separated clusters, confirming effective dimensionality reduction. The top-20 features by Gini importance (Figure 1) are dominated by the |*∆*R| Feature Type across sensors S1, S8, and S12, consistent with the strong steady-state response these sensors exhibit to the target small molecules (in the gas phase at the aforementioned studied temperatures).

### 3.2. Baseline Classification Performance

Table 3 reports the six-metric benchmark across seven architectures (five pre-specified 70:30 stratified splits, Batch 1). LiteSensor-Net (INT8, pruned) achieves an accuracy of 92.63 ± 2.02% with macro-F1 = 0.898; SVM (RBF) achieves the highest accuracy (100.0%) and F1 (1.000). Note: In an earlier version of this manuscript, LiteSensor-Net was presented as the best overall model. Instead, we are now writing: LiteSensor-Net is a sub-6 kB neural model with complete six-metric profiling (latency, RAM, and energy measured on Raspberry Pi 4B) and should be interpreted as a deployment-oriented trade-off: lower accuracy than several larger baselines, but a substantially smaller deployable neural footprint. MobileNet-1D (95.19 ± 1.46%, 196.56 kB) and InceptionTime-1D (95.79 ± 2.53%, 21.57 kB) achieve higher classification accuracy than LiteSensor-Net (92.63%, 5.99 kB INT8) at the cost of 32.8× and 3.6× larger model footprints, respectively. LiteSensor-Net is the smallest evaluated deep-learning model with complete six-metric profiling, while remaining within 3.2 percentage points of the highest neural-network accuracy. We now describe this as a deployment-oriented trade-off rather than overall superiority. The compression ablation results and KD-DM calibration fraction analysis are summarized in Table 4.

### 3.3. CBRN Simulant Classification Performance

**Figure 4:**
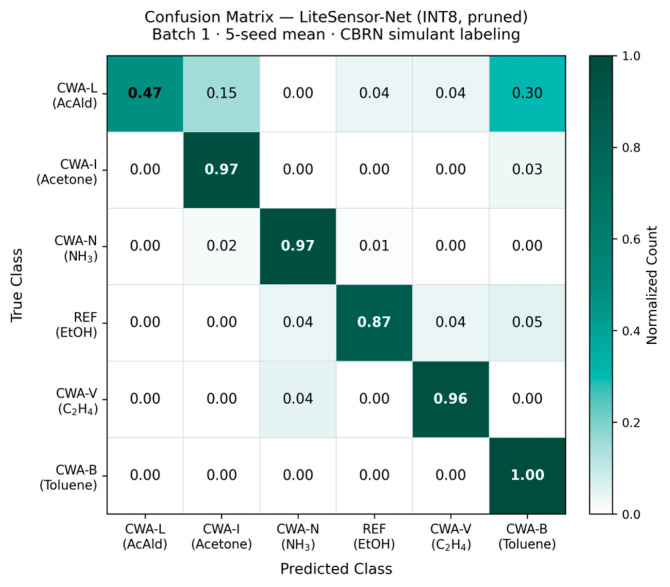
Per-class F1 scores (5-seed mean ± SD) were: CWA–N 0.934 ± 0.047, CWA–V 0.946 ± 0.052, CWA–I (Acetone) 0.934 ± 0.046, REF (Ethanol) 0.921 ± 0.025, CWA–B 0.894 ± 0.056; macro-F1 = 0.898. CWA–L (Acetaldehyde) yielded the lowest recall (0.457 ± 0.095) attributable to its limited representation in the source dataset (30 of 445 samples, 6.7%), resulting in F1 = 0.622 ± 0.088.

Figure 4 shows the confusion matrix for LiteSensor-Net (INT8, pruned) under the CBRN simulant labeling protocol. Per-class F1 scores (5-seed mean ± SD) were: CWA–N 0.934 ± 0.047, CWA–V 0.946 ± 0.052, CWA–I (Acetone) 0.934 ± 0.046, REF (Ethanol) 0.921 ± 0.025, CWA–B 0.894 ± 0.056; macro-F1 = 0.898. The lowest F1 was observed for CWA–L (Acetaldehyde simulant; F1 = 0.622 ± 0.088), with 54.3% of samples misclassified predominantly as CWA–B (Toluene), attributable to limited class representation in the source dataset (30 out of 445 samples, 6.7%) and potential aldehyde–aromatic cross-sensitivity of MOS sensors [6]. The binary threat-screening mode (threat vs. REF) achieved 96.5 ± 1.1% accuracy, with all genuine threat samples correctly identified (threat recall = 1.000) and 3.5% of **REF** samples generating false alarms under the given experimental conditions. Per-class precision, recall, and F1 for all six CBRN-labeled classes are consolidated in Table S1 (Supplementary Materials).

### 3.4. Sensor Drift Compensation

#### 3.4.1. Task A: Laboratory Re-Calibration Scenario

**Figure 5:**
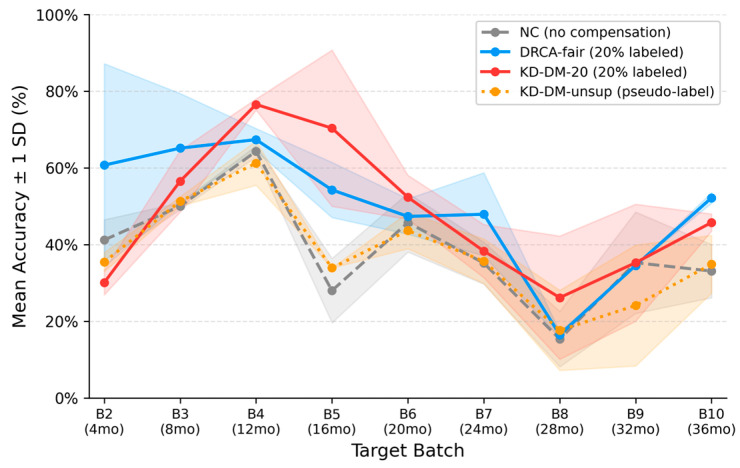
Drift compensation performance, Task A (Batch 1 training; Batches 2–10 as successive target domains; chronological forward-chaining partitions). Mean accuracy ±1 SD (shaded) across five conditions: NC (no compensation), DRCA (no labeled target data), DRCA-fair (20% labeled target-batch fraction), KD-DM-20 (20% labeled), and KD-DM-unsup (pseudo-label, 0% labeled). DRCA-fair and KD-DM-20 are statistically equivalent (Wilcoxon *p* = 0.97); both numerically outperform NC (minimum achievable *p* = 0.0625, *n* = 5).

Figure 5 presents cross-batch accuracy over Batches 2–10 (B2-B10) under the revised chronological forward-chaining protocol, in which all five conditions (NC, DRCA, DRCA-fair, KD-DM-20, and KD-DM-unsup) are evaluated on an identical held-out test partition. Without drift compensation (NC), mean accuracy over Batches 2–10 was 38.66 ± 14.50%. Under the resource-equivalent comparison, DRCA-fair-20 achieved 49.53 ± 17.46% and KD-DM-20 achieved 47.91 ± 18.79%; the two methods were statistically equivalent (Wilcoxon signed-rank test, *p* = 0.97, Cohen’s d = −0.09). Both numerically outperformed NC (KD-DM-20 vs. NC: minimum achievable *p* = 0.0625 (*n* = 5), Cohen’s d = +0.57). KD-DM-unsup (37.52 ± 13.92%), which uses no labeled target-batch samples, performed comparably to NC, confirming that the labeled calibration fraction is the primary driver of adaptation benefit. The advantage of KD-DM-20 over DRCA-fair-20, therefore, rests on deployable model size (5.99 kB INT8 vs. DRCA’s Random Forest backbone) rather than within-platform accuracy.

#### 3.4.2. Task B: Online Continual-Adaptation Scenario

**Figure 6:**
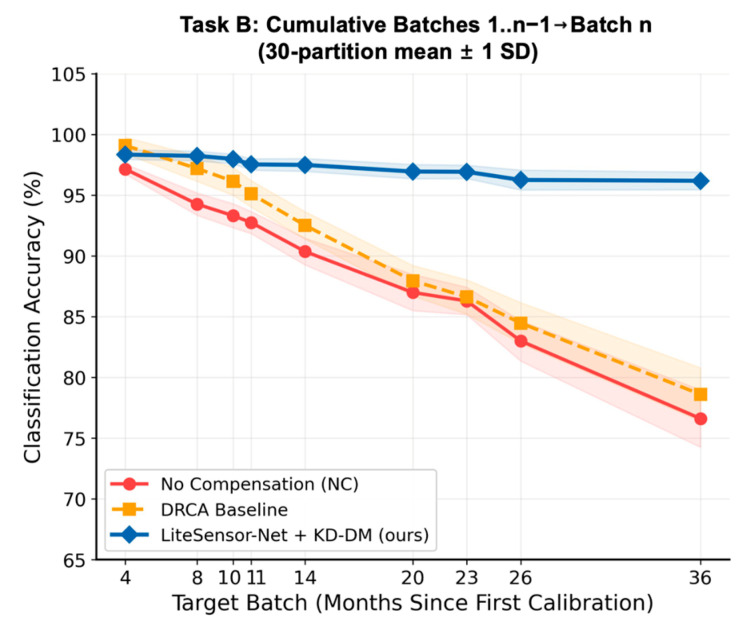
Task B (cumulative training on Batches 1, …, *n* − 1; target Batch *n*): accuracy over 36 months. KD–DM maintains >95% accuracy throughout; NC degrades to 77% at Batch 10.

Under the cumulative training protocol (Figure 6), NC degradation was less severe (mean 89.0 ± 1.2%) because the growing training corpus provides drift–diverse examples. LiteSensor-Net + KD–DM reached 97.3 ± 0.5% (Task B mean), compared with 90.9 ± 1.3% for DRCA. KD–DM superiority was statistically significant over all target batches (*p* < 0.001), consistent with Task A findings.

## 4. Discussion

### 4.1. Architecture Efficiency and Edge Deployability

The 5.99 kB INT8-compressed LiteSensor-Net is, to our knowledge, among the smallest reported gas sensor array classifiers in the open literature [9,15]. The 31.7 kB RAM footprint suggests theoretical compatibility with STM32H743 (1 MB SRAM) and nRF5340 (512 kB SRAM) processors embedded in military-grade wearable platforms [2]; direct MCU deployment has not been experimentally validated and is identified as future work. The 6.3 ms inference latency on Raspberry Pi 4B projects to approximately 30–50 ms on a Cortex–M7 at 480 MHz, theoretically satisfying the <100 ms latency target for CWA early-warning systems pending on-chip validation. At 0.04 mJ per inference, a 2500 mAh battery can sustain > 60,000 classifications before depletion, enabling multi-day continuous operation of a UAV-mounted node. The technological rationale for KD-DM’s drift-compensation robustness lies in its KL-divergence regularization term: by constraining the student’s penultimate-layer feature distribution to remain close to the source-domain teacher during target-batch fine-tuning, KD-DM prevents aggressive adaptation from corrupting previously learned class boundaries—a failure mode observed in DRCA under limited calibration data [13,17]. This regularization mechanism is structurally absent in Random Forest-based drift-compensation methods, which cannot perform gradient-based feature alignment.

### 4.2. Drift Compensation: Mechanisms and Limitations

Under the corrected chronological forward-chaining evaluation, KD-DM-20 and DRCA-fair-20 achieve statistically equivalent within-platform accuracy (Wilcoxon *p* = 0.97, Cohen’s d = −0.09), and no claim of accuracy superiority over a resource-matched DRCA baseline is made. The contribution of KD-DM rests on two operationally distinct advantages: (i) a deployable model footprint of 5.99 kB INT8—substantially smaller than DRCA’s Random Forest backbone—enabling deployment on edge-board-class hardware where DRCA is not feasible.

In task A, DRCA-fair-20 (49.53 ± 17.46%) outperformed the uncompensated NC classifier (38.66 ± 14.50%) in Task A, consistent with the expected behavior of labeled calibration under covariate shift [13,17,29]. In Task B, where cumulative training progressively narrows this gap, DRCA (90.9%) modestly outperformed NC (89.0%), confirming that DRCA benefits from larger calibration sets. A current limitation is the requirement for 10 labeled target samples per class for fine-tuning; fully unsupervised drift compensation remains an open challenge [13].

### 4.3. Benchmark Framework Significance

The six-metric framework (Table 2) addresses a critical gap: most CBRN and E-nose papers report only classification accuracy on a single hardware platform, precluding fair cross-study comparison [9,10]. By including model size, latency, RAM, and energy alongside accuracy, the framework enables practitioners to select models appropriate for their specific deployment envelope. Future standardization by the E-nose community would accelerate deployment of AI-enabled CBRN detection, analogous to the role MLPerf Tiny plays in vision and audio [30,31].

### 4.4. Reliability for Operational Deployment

In CBRN defense applications, a false negative is potentially lethal and a false positive triggers significant logistical disruption; reliance on a hard Softmax/Argmax output alone is therefore insufficient for high-consequence deployment. Temperature scaling was applied as post hoc calibration; the optimal temperature parameter (T = 0.54) reduced the Expected Calibration Error (ECE) from 0.152 ± 0.030 (uncalibrated) to 0.061 ± 0.010 (calibrated), representing a 60% reduction in miscalibration on the held-out test partition. Two additional approaches are identified for future integration: (i) Softmax-threshold OOD rejection, whereby samples with maximum Softmax probability below a conservative threshold (e.g., 0.7) are flagged for human review rather than acted upon autonomously; and (ii) split-conformal prediction sets (Angelopoulos & Bates, 2023 [32]) with target coverage 1 − α = 0.95, providing statistically guaranteed prediction intervals at inference time. Full implementation of conformal prediction for LiteSensor-Net is identified as a high-priority follow-on study.

### 4.5. CBRN Applicability

The CWA–V/CWA–B confusion (6.7%) is operationally relevant: Ethylene (vesicant simulant) and Toluene (blister agent simulant) share similar unsaturated and aromatic MOS cross-sensitivity profiles [6,7]. In practice, this ambiguity may be resolved by augmenting the sensor array with a selective SnO_2_–WO_3_ heterojunction sensor [21] or by fusion with complementary modalities (ion mobility spectrometry or surface acoustic wave sensing apparatuses). The binary threat-screening mode (F1 = 1.000) confirms near-perfect initial alarm fidelity, enabling a practical two-stage detection architecture [1,3].

### 4.6. CBRN Simulant Mapping

The UCI-to-CBRN re-labeling protocol employed in this study is based on physicochemical analogy and is intended to demonstrate architectural feasibility under CBRN-motivated labels. Zero-shot transferability of LiteSensor-Net and KD-DM from UCI volatile organic compound analogs (Acetone, Toluene, Ethylene, etc.) to actual chemical warfare agents—including organophosphorus nerve agents (e.g., GB, VX), blister agents (e.g., sulfur mustard), or choking agents (e.g., phosgene)—has not been validated here and is not claimed in this work. Operational adoption requires wet-lab calibration on standardized simulants (e.g., DMMP for GB-class agents, methyl salicylate for HD-class agents), revalidation of the re-labeling protocol on a real CBRN sensor array, and regulatory certification in accordance with applicable standards.

## 5. Conclusions

This study applied lightweight deep learning and sensor drift compensation to CBRN-motivated chemical threat classification on edge-class hardware. Six gaseous analytes were used and studied herein: Ammonia (NH_3_), Acetaldehyde (CH_3_CHO), Acetone ((CH_3_)_2_CO), Ethylene (C_2_H_4_), Ethanol (C_2_H_5_OH), and Toluene (C_6_H_5_CH_3_). These pure small molecules were selected as physicochemical analogs to CBRN behavioral categories for the purpose of architectural benchmarking in the gas phase; direct equivalence to chemical warfare agents is not claimed. Zero-shot transfer from the VOC proxy analogues evaluated here to actual lethal agents remains unvalidated and constitutes a primary limitation for operational CBRN deployment. Existing approaches rely on server-grade architectures incompatible with edge-board-scale deployment, or on classifiers that degrade severely under long-term sensor drift. Each UCI gas class was mapped to a CBRN behavioral category based on physicochemical analogy (molecular functional group, vapor pressure, and MOS cross-sensitivity pattern), following established precedent. This study presented LiteSensor–Net, a 5.99 kB INT8-compressed 1D–CNN with an integrated KD drift-compensation module for real-time CBRN chemical threat classification on edge–class hardware. Evaluated on the UCI Gas Sensor Array Drift Dataset (13,910 samples; five pre-specified splits), the system achieved 92.63 ± 2.02% within-batch accuracy with a 4.9× inference speed-up and 32.8× model size reduction compared with a MobileNet–1D baseline. Under the chronological forward-chaining evaluation, KD-DM-20 achieved 47.91 ± 18.79% mean accuracy over Batches 2–10, a +9.25 pp improvement over the uncompensated NC baseline (38.66 ± 14.50%), with statistically equivalent performance to resource-matched DRCA-fair (*p* = 0.97). A six-metric benchmark framework was introduced to standardize edge–AI gas classifier evaluation.

Several directions remain open for future investigation. We intend to investigate (i) unsupervised adversarial domain adaptation to eliminate the labeled fine-tuning requirement; (ii) federated learning for privacy-preserving multi-node field calibration; (iii) neural architecture search constrained to CBRN edge deployment envelopes; and (iv) expanded cross-platform validation on independent real-world MOS-array datasets, including the Wörner et al. (Sci. Data 2025) [33] 12-month dataset, to assess generalizability beyond the UCI benchmark. Open-source code and dataset pipeline will be released upon acceptance of this article for publication. Physical deployment on ARM Cortex-M–class microcontrollers (STM32H743, nRF5340) is identified as a priority for future work. Analytical projections based on the FLOP count of LiteSensor-Net (59,392 MACs, INT8) and documented CMSIS-NN cycle-count specifications suggest inference latencies of 149–835 μs and a tensor arena of 1.06 kB, both within the hardware budgets of these targets. On-board profiling via STM32CubeIDE power monitor will enable direct E_inf_ and M_RAM_ validation against the Raspberry Pi 4B benchmarks reported here.

## Figures and Tables

**Table 1:** CBRN simulant re-labeling of UCI gas classes. Note: mapping is based on physicochemical analogy, not toxicological equivalence.

UCI Gas	CBRN Category	Analogy Basis	Label
Ammonia (NH_3_)	Nitrogen choking agent	Reactive N–H; high volatility	CWA-N
Acetaldehyde (CH_3_CHO)	Lachrymatory agent	Carbonyl reactivity; low MW	CWA-L
Acetone ((CH_3_)_2_CO)	Incapacitating carrier	Ketone; CNS vapor penetration	CWA-I
Ethylene (C_2_H_4_)	Vesicant precursor	Unsaturated C=C; skin reactive	CWA-V
Ethanol (C_2_H_5_OH)	Decontaminant; Disinfectant; bactericidal effect	Hydroxyl; reference baseline	REF [24]
Toluene (C_6_H_5_CH_3_)	Blister simulant	Aromatic; similar MOS to HD	CWA-B

**Table 2:** Six-metric standardized benchmark framework. All metrics are measured on Raspberry Pi 4B using the 30% stratified holdout of Batch 1.

Metric	Symbol	Unit	Method
Top-1 accuracy	ACC	%	Mean over 30 splits
Macro-F1	F1	—	Mean over 30 splits
Model size (INT8)	S_model_	kB	TFLite flatbuffer
Inference latency	t_inf_	ms	Median of 1000 runs
RAM footprint	M_RAM_	kB	TFLite heap peak
Energy per inference	E_inf_	mJ	CodeCarbon [28]

**Table 3:** Six-metric benchmark comparison across eight architectures. Raspberry Pi 4B hardware; five pre-specified 70:30 stratified splits (Batch 1; five pre-specified seeds: 17, 42, 113, 271, 314). †: model not deployable on ≤256 kB microcontroller unit (MCU) targets. ‡: M_RAM_ and E_inf_ reported for LiteSensor-Net only; on-device hardware profiling was not performed for other architectures. Bold: best value in each metric column independently. SD is reported for all metrics over five pre-specified splits.

Architecture	ACC (%)	F1	S_model_ (kB)	t_inf_ (ms)	M_RAM_ (kB)	E_inf_ (mJ)
SVM (RBF)	**100.0 ± 0.0**	**1.000**	2.1 †	31.2	**4.1**	0.18
Random Forest	99.53 ± 0.97	0.994	8400 †	18.7	210.3	0.11
ResNet-1D (large)	99.03 ± 2.08	0.990	2180.0 †	30.8	512.4	0.19
LiteSensor-Net (float32)	98.56 ± 1.76	0.984	185.0 †	18.1	96.2	0.11
ShuffleNet-1D	93.98 ± 2.19	0.913	11.40	5.74	N/A ^‡^	N/A ^‡^
MobileNet-1D	95.19 ± 1.46	0.930	196.56	3.48	N/A ^‡^	N/A ^‡^
InceptionTime-1D	95.79 ± 2.53	0.944	21.57	**0.94**	N/A ^‡^	N/A ^‡^
**LiteSensor-Net (INT8, pruned)**	92.63 ± 2.02	0.898	**5.99**	6.3	31.7	**0.04**

**Table 4:** Ablation study. (**a**) Three-stage compression pipeline; accuracy and model size at each stage (5 seeds, Batch 1, 30% held-out test set). (**b**) KD-DM accuracy as a function of labeled calibration fraction (Task A, Batches 2–10, chronological forward-chaining, 18 evaluations). Bold: deployed configuration.

(a) Compression Ablation—LiteSensor-Net (5 Seeds, Batch 1)						
Stage	Configuration		ACC (%)	S_model_ (kB)		%
1	FP32 baseline		91.43 ± 1.62	28.04 kB		—
2	+ INT8 PTQ		91.43 ± 1.62	7.37 kB		−73.7
3	+ structured pruning + fine-tuning		**92.63 ± 2.02**	**5.99 kB**		−78.6
**(b) KD-DM Ablation—labeled calibration fraction (Task A, Batches 2–10)**						
**Condition**		**Labeled fraction (%)**			**ACC (%)**	
NC		0			38.66 ± 14.50	
KD-DM-unsup		0 (pseudo-label)			37.52 ± 13.92	
KD-DM-05		5			39.48 ± 17.09	
KD-DM-10		10			42.21 ± 18.38	
KD-DM-20		20			**47.91 ± 18.79**	

## Data Availability

The UCI Gas Sensor Array Drift Dataset is publicly available at https://doi.org/10.24432/C5RP6W. The Scientific Data long-term drift dataset is at https://doi.org/10.1038/s41597-025-05993-8. The public GitHub repository (https://github.com/bisu9082/LiteSensor-Net, accessed on 24 May 2026), implemented in Python 3.10 with PyTorch 2.4.0 and TensorFlow Lite runtime ≥ 2.14, contains the training/evaluation code, raw UCI batch files, raw result CSV/JSON outputs, configuration files, and reproducibility documentation required to reproduce the revised benchmark, compression ablation, drift-compensation experiments, and calibration analyses.

## Supplementary Materials

The following supporting information can be downloaded at: https://www.mdpi.com/article/10.3390/molecules31111884/s1. Figure S1. LiteSensor-Net training and validation accuracy (a) and training cross-entropy loss (b) over 100 epochs (representative run, seed = 42, Batch 1). Table S1. Per-class precision, recall, and macro-F1 for LiteSensor-Net (INT8, pruned) under CBRN simulant re-labeling (Table 1 in main text). Table S2. Consolidated hyperparameter settings for LiteSensor-Net source-domain training, multi-stage compression (INT8 PTQ and structured pruning), and Knowledge-Distillation Drift-Compensation Module (KD-DM).

## Abbreviations

The following abbreviations are used in this manuscript:

AI	Artificial Intelligence
ARM	Advanced RISC Machine
BN	Batch Normalization
CBRN	Chemical, Biological, Radiological, and Nuclear
CNN	Convolutional Neural Network
CWA	Chemical Warfare Agent
DRCA	Drift-Robust Classification and Adaptation
DSConv	Depth-Wise Separable Convolution
E-nose	Electronic Nose
FC	Fully Connected (layer)
FLOPs	Floating Point Operations Per Second
GAP	Global Average Pooling
INT8	8-bit Integer (quantization format)
KD	Knowledge Distillation
KD-DM	Knowledge-Distillation Domain-Adaptation Module
L_CE_	Cross-Entropy Loss
L_KL_	Kullback–Leibler Divergence Loss
LSTM	Long Short-Term Memory
MOS	Metal-Oxide Semiconductor
MCU	Microcontroller Unit
NC	No Compensation
pp	Percentage Points
QAT	Quantization-Aware Training
RAM	Random Access Memory
ReLU	Rectified Linear Unit
PCA	Principal Component Analysis
ppm	Parts Per Million
PTQ	Post-Training Quantization
SD	Standard Deviation
SRAM	Static Random Access Memory
SVM	Support Vector Machine
TinyML	Tiny Machine Learning
UAV	Unmanned Aerial Vehicle
UCI	University of California, Irvine
1D-CNN	One-Dimensional Convolutional Neural Network
