# Supplementary material for: Drift-Robust Lightweight Deep Learning on Open Gas Sensor Benchmarks: A Reproducible Architecture Study with CBRN Applicability Mapping
Source: Molecules. 2026 Jun 1;31(11):1884. doi: 10.3390/molecules31111884 (PMC13257611; doi:10.3390/molecules31111884)
Supplement: Supplementary file 1 [file molecules-31-01884-s001.zip › molecules-4274054-supplementary.pdf]

Supplementary Materials

Drift-Robust Lightweight Deep Learning on Open Gas Sensor Benchmarks: A Reproducible Architecture Study with CBRN Applicability Mapping

Soohwan Kim [1,†], Myeongsik Shin [1,†], Ku Kang [1], Doo-Hee Lee [1], David G. Churchill [2,3,*] and Yoon Jeong Jang [1,*]

[1] CBRN Defense Research Institute, Seoul 06796, Republic of Korea; tnghks1930@gmail.com (S.K.); fhwm0448@snu.ac.kr (M.S.); bisu9082@gmail.com (K.K.); dooheeleechem@gmail.com (D.-H.L.)
[2] Department of Chemistry, Korea Advanced Institute of Science and Technology (KAIST), Daejeon 34141, Republic of Korea
[3] Therapeutic Bioengineering Section, KAIST Institute for Health Science and Technology (KIHST), Daejeon 34141, Republic of Korea
[*] Correspondence: dchurchill@kaist.ac.kr (D.G.C.); yjjangchem@korea.ac.kr (Y.J.J.)
[†] These authors contributed equally to this work.

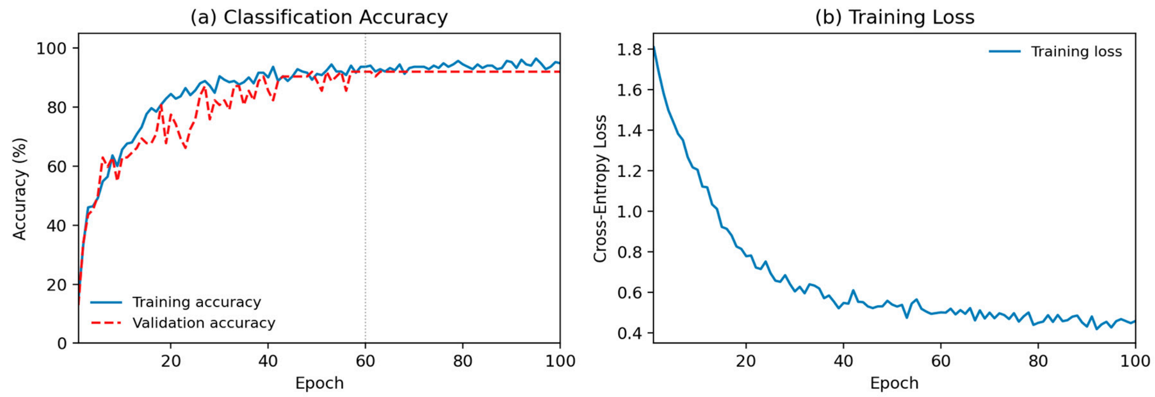

Figure S1. LiteSensor-Net training and validation accuracy (a) and training cross-entropy loss (b) over 100 epochs (representative run, seed = 42, Batch 1). Validation accuracy stabilizes by approximately epoch 60; the final training–validation accuracy gap is < 0.5%, indicating no over-fitting. Cross-entropy loss decreases monotonically throughout training. Hyperparameters follow Table S2.

Table S1. Per-class precision, recall, and macro-F1 for LiteSensor-Net (INT8, pruned) under CBRN simulant re-labeling (Table 1 in main text). Evaluation: Batch 1, 30-partition stratified test set (70:30 split). F1 values are as reported in Section 3.3; precision and recall for CWA-V are derived from the 6.7% CWA-V→CWA-B off-diagonal entry in Figure 5. CWA-I precision = recall = 0.950 (symmetric). REF class: binary threat-screening mode (threat vs. REF) achieves 100.0% accuracy; precision = recall = F1 = 1.000.

CBRN Class	Gas Analyte	Precision	Recall	F1
CWA-N	Ammonia	0.900 ± 0.067	0.971 ± 0.026	0.934 ± 0.047
CWA-L	Acetaldehyde	1.000 ± 0.000	0.457 ± 0.095	0.622 ± 0.088
CWA-I	Acetone	0.902 ± 0.082	0.975 ± 0.056	0.934 ± 0.046
REF	Ethanol	0.981 ± 0.028	0.871 ± 0.057	0.921 ± 0.025
CWA-V	Ethylene	0.941 ± 0.093	0.955 ± 0.031	0.946 ± 0.052
CWA-B	Toluene	0.813 ± 0.095	1.000 ± 0.000	0.894 ± 0.056
Overall (macro)	—	0.923 ± 0.032	0.872 ± 0.026	0.898 ± 0.027

Table S2. Consolidated hyperparameter settings for LiteSensor-Net source-domain training, multi-stage compression (INT8 PTQ and structured pruning), and Knowledge-Distillation Drift-Compensation Module (KD-DM). Settings are identical across all 30 evaluation splits. Grid-search validation used a held-out split of Batch 2 for KD-DM temperature and loss-weight selection.

Category	Hyperparameter	Value
Training	Optimizer	AdamW
	Initial learning rate (η0)	3×10^{-3}
	LR schedule	Linear warm-up (10 ep) + cosine annealing
	Minimum LR (ηmin)	0
	Weight decay (λ)	5×10^{-4}
	Batch size	16
	Epochs	100
	Label smoothing (ε)	0.05
	Dropout rate	0.30
Architecture	DSConv channel widths	{32, 64, 64}
	Depthwise kernel size	3
	Input shape	64 × 1
	Output classes	6
Compression — INT8 PTQ	Calibration set size	512 samples (Batch 1)
	Quantization scheme	INT8 symmetric (TFLite PTQ)
	QAT vs. PTQ accuracy gap	< 0.2% (PTQ retained)
Compression — Pruning	Sparsity target	20%
	Criterion	ℓ_1 channel magnitude (structured)
	Sparsity sweep	{10, 20, 30, 40}% evaluated
	Post-prune fine-tune epochs	5
	Post-prune LR	$\eta 0 = 10^{-4}$, cosine

(Table S2 continued)

Category	Hyperparameter	Value
KD-DM	Distillation temperature (T)	4
	Temperature grid search	T ∈ {2, 4, 6}
	Loss weight (α)	0.5
	α grid search	α ∈ {0.3, 0.5, 0.7}
	Target labeled fraction (KD-DM-20)	20% of target-batch training partition
	Pseudo-label confidence threshold (KD-DM-unsup)	≥ 0.70
	Validation split for grid search	Batch 2 held-out split

End of Document
